# Comparative analysis of the kinomes of three pathogenic trypanosomatids: *Leishmania major*, *Trypanosoma brucei *and *Trypanosoma cruzi*

**DOI:** 10.1186/1471-2164-6-127

**Published:** 2005-09-15

**Authors:** Marilyn Parsons, Elizabeth A Worthey, Pauline N Ward, Jeremy C Mottram

**Affiliations:** 1 Seattle Biomedical Research Institute, 307 Westlake Ave. N., Seattle, WA, 98109 USA; 2 Department of Pathobiology, University of Washington, Seattle, WA, 98195 USA; 3 Wellcome Centre for Molecular Parasitology, The Anderson College, University of Glasgow, Glasgow G11 6NU, UK

## Abstract

**Background:**

The trypanosomatids *Leishmania major*, *Trypanosoma brucei *and *Trypanosoma cruzi *cause some of the most debilitating diseases of humankind: cutaneous leishmaniasis, African sleeping sickness, and Chagas disease. These protozoa possess complex life cycles that involve development in mammalian and insect hosts, and a tightly coordinated cell cycle ensures propagation of the highly polarized cells. However, the ways in which the parasites respond to their environment and coordinate intracellular processes are poorly understood. As a part of an effort to understand parasite signaling functions, we report the results of a genome-wide analysis of protein kinases (PKs) of these three trypanosomatids.

**Results:**

Bioinformatic searches of the trypanosomatid genomes for eukaryotic PKs (ePKs) and atypical PKs (aPKs) revealed a total of 176 PKs in *T. brucei*, 190 in *T. cruzi *and 199 in *L. major*, most of which are orthologous across the three species. This is approximately 30% of the number in the human host and double that of the malaria parasite, *Plasmodium falciparum*. The representation of various groups of ePKs differs significantly as compared to humans: trypanosomatids lack receptor-linked tyrosine and tyrosine kinase-like kinases, although they do possess dual-specificity kinases. A relative expansion of the CMGC, STE and NEK groups has occurred. A large number of unique ePKs show no strong affinity to any known group. The trypanosomatids possess few ePKs with predicted transmembrane domains, suggesting that receptor ePKs are rare. Accessory Pfam domains, which are frequently present in human ePKs, are uncommon in trypanosomatid ePKs.

**Conclusion:**

Trypanosomatids possess a large set of PKs, comprising approximately 2% of each genome, suggesting a key role for phosphorylation in parasite biology. Whilst it was possible to place most of the trypanosomatid ePKs into the seven established groups using bioinformatic analyses, it has not been possible to ascribe function based solely on sequence similarity. Hence the connection of stimuli to protein phosphorylation networks remains enigmatic. The presence of numerous PKs with significant sequence similarity to known drug targets, as well as a large number of unusual kinases that might represent novel targets, strongly argue for functional analysis of these molecules.

## Background

Trypanosomatid pathogens of humans include *Trypanosoma brucei*, *Trypanosoma cruzi *and *Leishmania major*, causative agents of African sleeping sickness, Chagas disease, and cutaneous leishmaniasis respectively [1]. *Trypanosoma brucei *lives extracellularly in the human host, primarily in the bloodstream and cerebrospinal fluid. African sleeping sickness, which is estimated to afflict 300,000–500,000 people per year in sub-Saharan Africa, with a disease burden of 1.6 million disability adjusted life years (DALYs), is invariably fatal unless treated [2]. *Trypanosoma cruzi*, which is found in Latin America, results in a disease burden of 650,000 DALYs. This parasite can invade most types of nucleated cells. About 30% of infected individuals progress to a chronic phase that culminates in heart disease and mega syndrome [3]. Of those infected it is estimated the 50,000 will die each year. *Leishmania *parasites result in a disease burden of 2.3 million DALYs, with greater than 80,000 deaths/year and cause a variety of diseases depending on the infecting species. The most dangerous manifestation is the visceral disease known as kala azar, caused by *L. donovani*. Kala azar is re-emerging in India in a particularly aggressive form that is resistant to standard treatment [4]. No vaccine has been approved for any of these diseases and many of the drugs in use are highly toxic and prone to the development of drug resistance. There is therefore an urgent need to identify new drug targets and the recent completion of the genome sequence of the three model trypanosomatids, *T. brucei*, *T. cruzi *and *L. major*, can be exploited in this regard [5-7].

During development the parasites pass through different environments. Each species is carried by a different insect vector, in which the parasite undergoes specific developmental changes that allow it to infect the human host. For example, *Leishmania *parasites move from the sandfly midgut up to the mouthparts, then into the human host where they invade macrophages and live within a phagolysosome. In each environment, the parasites respond with significant changes in their metabolic and protein profile. The signal transduction pathways mediating these changes remain unknown. Only a few receptor-like proteins have been identified, primarily receptor adenylate cyclases with an extracellular putative ligand binding domain and an intracellular catalytic domain [8,9]. Intermediate steps of signal transduction in the parasites have not been defined, although genomic analysis shows that they possess numerous molecules predicted to bind second messengers, as well as protein kinases and phosphatases [5]. The culmination of the signaling pathways is unlikely to be at the level of transcription, since most genes are transcribed in polycistronic units with little evidence for regulation [7,10,11]. Many changes in protein phosphorylation during the parasite developmental cycles have been documented [12-14]. The parasites also possess an integrated cell cycle that coordinates the inheritance of the single mitochondrion, flagellum, and nucleus [15,16].

Protein kinases (PKs) are key mediators of signal transduction, transmitting environmental cues and coordinating intracellular processes. Eukaryotic protein kinases (ePKs) are categorized by the amino acid sequence of their catalytic domains. Broadly, ePKs fall into two superfamilies: protein serine/threonine kinases and protein tyrosine kinases. The former are ubiquitous in eukaryotes. The latter are present in all metazoa for which the genome sequence is available, but relatively few examples have been found in unicellular eukaryotes [17,18]. However, protein tyrosine phosphorylation has been well documented in trypanosomatids [13,14,19,20]. Mammalian receptor protein kinases are generally tyrosine kinases [21], while all known plant receptor kinases are serine/threonine kinases [22,23]. Receptor kinases are activated by ligands, facilitating intercellular communication within multicellular organisms. Parasites that live in multicellular hosts could conceivably use similar mechanisms to respond to host or parasite ligands, although such reactions have not been defined at the molecular level.

Six major groups of ePKs have been defined on the basis of sequence similarity of the catalytic domains: AGC, CAMK, CMGC, TK, TKL, STE [24]. ePKs that do not fall into these groups are categorized as "Other". Within each group (including "Other"), multiple families have been defined. Interestingly, the substrate preferences break into groups along the same lines: for example AGC and CAMK kinases tend to phosphorylate motifs containing basic residues, CMGC kinases often are proline-directed, while CK1 and CK2 kinases phosphorylate motifs with acidic residues [24]. Additional features that correlate with group assignments include responses to other mediators such as ligands (receptor TK), calcium (CAMK), and certain second messengers (AGC). Relatively few protein kinases have been studied in detail with respect to expression and function in each of the trypanosomatids (see Additional file 1).

Atypical PKs (aPKs) are not closely related to ePKs at the sequence level, lacking the 11 subdomains that define ePKs. They include a variety of molecules that have been shown to have protein kinase catalytic activity in specific systems. Among the aPKs, the most well-characterized are the PIKK kinases, which have catalytic domains resembling those of lipid kinases in sequence [25]. Interestingly, the RIO and alpha groups show remnants of many of the ePK subdomain motifs [26,27]. The other atypical kinases require further study for definitive analysis of their activity.

An analysis of partial genomic sequence suggested that trypanosomatids might differ considerably from the host in signaling mechanisms, lacking typical signaling receptors with the exception of adenylate cyclases, as well as SH2 domains and transcription factors [28]. These speculations have been borne out by the completed genomic sequences of the three trypanosomatids known as the TriTryps: *T. brucei *[6], *T. cruzi *[5], and *L. major *[7], as briefly discussed in the *T. cruzi *genome paper [5]. In this report, we present a detailed examination of the TriTryp kinome.

## Results and discussion

### The TriTryp kinome

To identify all protein kinase genes in the three trypanosomatid genomes, we searched GeneDB [29] for all genes bearing Pfam protein kinase domains, as well as by BLAST using representatives of all major protein kinase gene families, including aPKs. All ePKs were examined for the presence of the 11 characterized subdomains, and specifically for the presence of the key lysine in subdomain 2 and aspartic acids in subdomains 6 and 7. The genomic analysis revealed 179, 156, and 171 ePKs and 17, 20, 19 aPKs in *L. major*, *T. brucei *and *T. cruzi *respectively. These numbers suggest that phosphorylation is an important mechanism for cellular regulation in all three trypanosomatids and are considerably larger than that described for another intracellular parasite that transits diverse environments; *Plasmodium falciparum*. *P. falciparum *possesses 65 ePKs and 20 ePK-related sequences, designated FIKK [30-32]. The latter have not yet been shown to have protein kinase activity [30]. The activation of many ePKs requires phosphorylation in the activation loop between subdomains 7 and 8. These kinases are typically marked by an RD motif within subdomain 6 [33]. In *T. brucei*, 130 of the 156 ePKs are RD kinases, further supporting the concept that phosphorylation networks are complex and important in these organisms.

**Figure 1:**
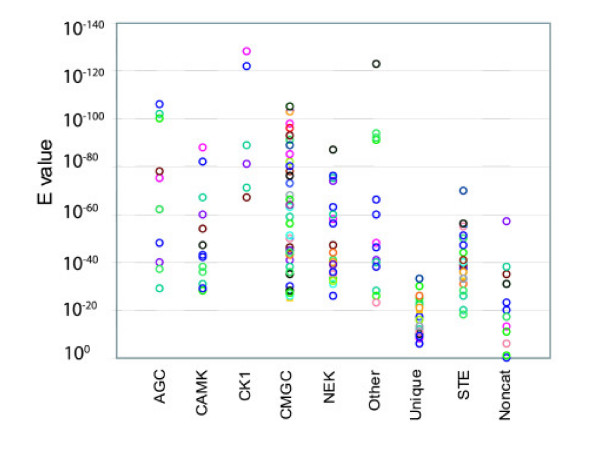
**Similarity of *T. cruzi *ePKs to those in the 4-kinome dataset**. Full-length proteins were tested by BLAST analysis against the database of catalytic domains of all human, *Saccharomyces cerevisiae*, *Drosophila melanogaster*, and *Caenorhabditis elegans *protein kinases. The best E-value is graphed for each kinase, which are clustered according to their classification group. Non-cat: protein kinases predicted to be non-catalytic due to lack of subdomain I or catalytic residues. Different colors were used to facilitate viewing of closely spaced spots.

**Figure 2:**
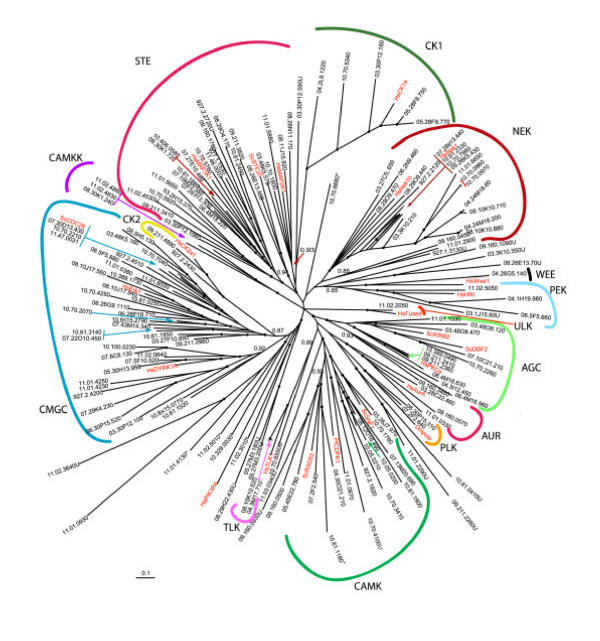
**Unrooted tree of *T. brucei *protein kinases**. The catalytic domains of predicted functional ePKs were analyzed using MRBAYES. Bootstrap values greater than 0.95 are indicated by a dot at the node, and selected lower values are shown. No members clustered with TK or TKL kinases from human or yeast (not shown on the tree). *T. brucei *sequences are identified by systematic gene IDs. Selected human (Hs), *S. cerevisiae *(Sc) and *D. melanogaster *(Dm) were included to provide landmarks and these are shown in red font. Asterisks mark sequences for which the MRBAYES tree conflicted with the BLAST analysis at the group level. Kinases classified as unique through BLAST are marked with "U".

We examined the relationship of the trypanosomatid ePKs to the groups and families of kinase domains of human, worm, fly, and yeast using the available datasets [34]. Most ePKs had a highly significant BLAST score against at least one member of the 4-kinome dataset. For example, 58% of the *T. cruzi *ePKs had an E-value of at least 10^-40^, and 77% had a score of at least 10^-30 ^against a member of this dataset (Figure 1). Based on BLAST E-values, as well as phylogenetic inference (see below), assignments to ePK groups and families were made (Table 1, Additional file 1). We generated phylogenetic trees of the kinase domains of the entire *T. brucei *ePK kinome, seeding the tree with human and yeast PKs to facilitate classification (Figure 2 shows the MRBAYES tree). The trees were generally consistent with the BLAST assignments (the few that did not match are marked by an asterisk in the tree). Of note are several unique kinases that are on long branches originating near the center of the tree, indicating their high divergence from other ePKs.

**Table 1:** Groups and families of ePKs in the TriTryps

**Group**	**Family**	***T. brucei***	***T. cruzi^a^***	***L. major***	**Group**	**Family**	***T. brucei***	***T. cruzi***	***L. major***
**AGC**	(na)^b^	6	7	6	**Other**	AUR	3	3	3
	NDR	2	1	1		CAMKK	4	4	4
	PKA	3	3	3		CK2	2	2	2
	RSK	1	1	1		NAK	0	1	1
	**Total**	**12**	**12**	**11**		NEK	20	22	22
					
**CAMK**	(na)	7	7	9		PEK	3	2	3
	CAMKL	7	6	7		PLK	2	2	1
	**total**	**14**	**13**	**16**		TLK	2	1	1
					
**CK1**	CK1	4	7	6		ULK	1	1	1
	TTBK	1	1	1		VPS	1	1	0
	**Total**	**5**	**8**	**7**		WEE	1	2	2
					
**CMGC**	(na)	1	1	1		**total**	**39**	**42**	**40**
					
	CDK	11	10	11	**STE**	(na)	7	8	10
	CLK	4	5	4		STE7	2	2	2
	DYRK	7	7	8		STE11	14	18	21
	GSK	2	2	2		STE20	2	3	1
	CDKL (MAPK-like)^c^	2	1	2		**total**	**25**	**31**	**34**
					
	MAPK^d^	10	11	12	**Unique**		**19**	**23**	**26**
					
	RCK (MAPK-like)^c^	3	3	3	**TOTAL ePK**		**156**	**171**	**179**
	SRPK	2	2	2					
					
	**total**	**42**	**42**	**45**	**Non-catalytic**		**13**	**16**	**16**

^a ^Multiple copies of TcCRK7 were counted as one gene. The number of tandem repeated CK1 genes in *T. cruzi *is not clear. One putative *T. cruzi *PEK gene is truncated by the contig end for both alleles, and is assigned on the basis of orthology in the non-catalytic sequence.

^b^na, not assigned to a family.

^c ^CDKL and RCK families are related to MAPKs and CDKs [57] but are classified separately in the human kinome [21].

^d ^One MAPK-like sequence in *T. brucei *and two in *T. cruzi *and *L. major *lack the typical regulatory motifs (see Table S1). One MAPK-like COG, which fell below the E-value criterion, was designated as MAPK on the basis of regulatory motifs.

**Figure 3:**
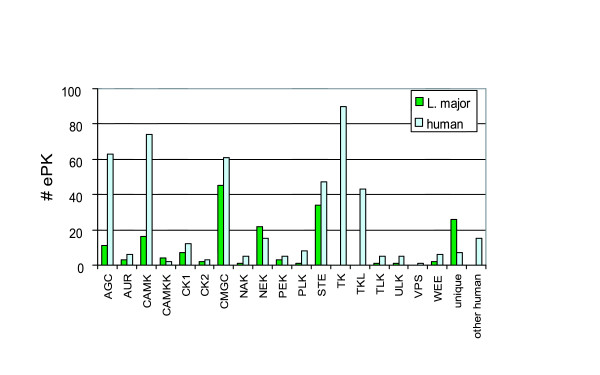
Comparison of *L. major *and human ePK classification.

In general, the TriTryp kinomes are closely related. COGS are **c**lusters of **o**rthologous **g**enes, as revealed by analysis of mutual BLASTP hits across the genomes. The majority (68%) of the ePK genes reside in COGS that contain members from each of the three species (Additional file 1). Conversely, only a small number of genes appear to be unique to a species (20 in *L. major*, 11 in *T. cruzi*, and 3 in *T. brucei*, Additional file 1). As with other genes in these organisms, members of these COGs are generally syntenic among the three species, furthering the concept that the molecules are orthologous. Figure 3 compares the representation in the major groups and families of human protein kinases with those of *L. major *and Table 1 shows the representation of groups and families among the TriTryps, with further details including systematic gene names provided in Additional file 1.

### Protein tyrosine kinases

A key difference between host and parasite kinomes is the complete lack of ePKs that map to the tyrosine kinase (TK) and tyrosine kinase-like (TKL) groups in the trypanosomatids. Representatives of the former in humans include receptor protein kinases such as the insulin receptor and cytosolic kinases such as src. The latter group contains ePKs such as RAF1 and TGFβR2. We also found no evidence of the receptor guanylyl cyclase (RGC) group of proteins, which are structurally related to protein kinases. These groups of ePKs are also absent in malaria parasites, which further lack the STE group of kinases. Interestingly, it has been reported recently that the genome of the unicellular protist *Entamoeba histolytica *encodes TKs with SH2 domains, TKLs, and a large family of putative receptor serine-threonine ePKs [18]. As *E. histolytica *also possesses genes encoding putative 7 transmembrane receptors and heterotrimeric G proteins, which the trypanosomatids lack [5], the mechanisms regulating cell signaling appear to be very different among the parasitic protozoa.

As noted above, phosphorylation on tyrosine is well documented in trypanosomatids. We propose that this activity is likely to be due to the action of atypical tyrosine kinases such as Wee1 and dual-specificity kinases that can phosphorylate serine, threonine, and tyrosine. Multiple members of the dual specificity kinase families (DYRKs, CLKs, and STE7) are present in the trypanosomatid genomes. Although Wee1 is functionally a tyrosine kinase, it most closely resembles serine/threonine kinases such as Chk1 and cAMP-dependent kinases in structure and primary amino acid sequence [35]. In yeast and higher eukaryotes Wee1 phosphorylates a conserved tyrosine residue in the ATP binding pocket of CDK1 (cdc2), inactivating the protein kinase. This mechanism is likely to be conserved in the three trypanosomatids, since there are two Wee1 family members in *L. major *and *T. cruzi *and one in *T. brucei*. In addition, CRK3, the putative functional CDK1 homologue in trypanosomatids [36-38], contains a conserved tyrosine residue in the same subdomain as the human CDK1 regulatory tyrosine [39,40]. In *T. brucei*, 18 other CMGC members also have this tyrosine within subdomain 1 (Additional file 2), reiterating the potential for widespread regulation of protein kinase activity via tyrosine phosphorylation. The potential conservation in regulatory mechanisms for CDK activity between yeast, mammals and trypanosomatids may not extend to all protozoa, as the putative *P. falciparum *Wee1 lacks a key active site residue suggesting it may not be active [31] and dual-specificity ePKs appear to be absent. In addition, no tyrosine phosphorylation has been demonstrated to date in that species. The existence of other unusual protein tyrosine kinases in trypanosomatids is an intriguing possibility given the large number of protein kinases in the trypanosomatid kinomes that cannot be easily placed into typical ePK groups or families (see below).

### Serine-threonine protein kinases

#### Poorly represented groups: CAMK and AGC

**Figure 4:**
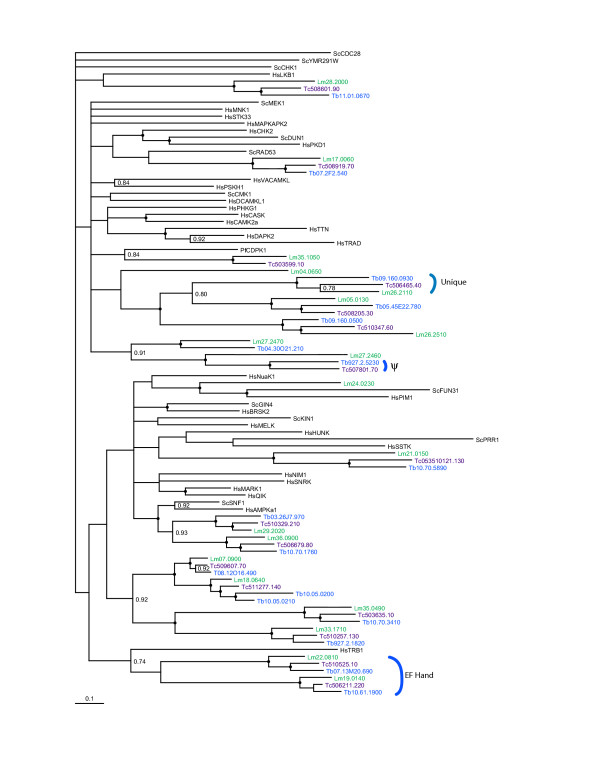
**Phylogram of CAMK ePKs**. Kinase domains from the TriTryp predicted proteins classified as CAMKs by BLAST or CAMK-like from the *T. brucei *tree in Figure 2 were analyzed using MRBAYES. Also included are CAMKs from all families present in humans (Hs), plus several *S. cerevisiae *(Sc) and a *P. falciparum *(Pf) kinase. Nodes with a bootstrap value greater than 0.95 are marked by a dot, while values ranging from 0.7–0.95 are indicated numerically. Similar results were obtained with PAUP*. Kinase domains are indicated by systematic gene IDs, which are abbreviated in the case of *L. major *to Lm *in lieu *of LmjF. Additionally, the invariant digits in the *T. cruzi *systematic names were deleted (all gene names start with Tc00.1047053). Only one *T. cruzi *allele was included for each gene. ψ marks a *T. cruzi *and *T. brucei *gene that are predicted to be non-functional due to a lack of a recognizable subdomain 1. The CAMK-like kinases classified as unique by BLAST are marked, as are the trypanosomatid CAMKs with EF-hand accessory domains. ScCDC28, a CMGC kinase, was used as an outgroup.

The CAMK and AGC groups are relatively poorly represented within trypanosomatid genomes as compared to humans. The CAMK group (which includes the Ca^+2^/calmodulin regulated kinases and AMP-dependent kinase, AMPK) is small in trypanosomatids with only 13 CAMKs predicted to be active in *T. cruzi*, 14 in *T. brucei*, and 16 in *L. major*. In contrast, the human genome encodes 74 CAMKs. A phylogenetic tree of the trypanosomatid CAMK and CAMK-like unique kinase domains is shown in Figure 4. Also included are along with representatives of each family of CAMKs from human, two yeast CAMKs and a plasmodial calcium dependent CAMK. Of the 19 trypanosomatid CAMK genes identified, 13 have representatives in each species as determined by COG analysis, and supported by phylogenetic trees. An additional CAMK-like kinase, marked as unique due to its low E-value in BLAST analysis, was also conserved, as was a COG in which two of the three orthologues were predicted to be inactive. The tree shown in Figure 4 also shows a characteristic common to all trees in which groups of ePKs from the trypanosomatids were compared: the trypanosomatid ePKs falling within COGS formed a tight cluster with high confidence, and were more distantly related to ePKs from humans or yeast.

BLAST analysis against the 4-kinome dataset indicated that about half of the trypanosomatid CAMKs belong to the CAMKL subfamily, the remainder were not assigned to a specific family. The phylogenetic trees generally agreed with these predictions, and supported the further classification of two sets of trypanosomatid genes as members of the AMPK subfamily. In other organisms, AMPKs are regulated by AMP and hence are involved in metabolic sensing [41]. In addition, two sets of the predicted CAMK kinases contain EF hand sequences, which may provide for sensitivity to Ca^+2 ^(marked in Figure 4). This juxtaposition of a protein kinase domain with EF hand motifs is characteristic of CDPKs, a group of calcium dependent protein kinases that are prominent in plants [42] and in *P. falciparum *[30,31,43], but which are absent in humans and yeast. However, the phylogenetic inference does not support the clustering of these trypanosomatid CAMKs with the plasmodial calcium dependent kinase CDPK1. These trypanosomatid genes are likely therefore to encode a novel class of EF-hand containing ePKs.

The AGC group includes ePKs structurally related to protein kinases that respond to second messengers: protein kinase A (responsive to cAMP), protein kinase G (responsive to cGMP), and protein kinase C (responsive to diacyl glycerol). Normalized to kinome size, trypanosomatids have approximately half as many AGC kinases as humans. The parasite genomes encode 3 AGC kinases that are related to PKA. However, *T. brucei *PKA appears to be activated by cGMP rather than cAMP [44]. Also within the AGC group are the NDR kinases. BLAST analysis and phylogenetic tree inference indicates that *T. brucei *possesses two NDR family kinases. One of these is conserved and syntenic among the trypanosomatids, while the other, PK50 (Tb10.70.2260), is specific to *T. brucei*. This molecule is a functional homologue of *Schizosaccharomyces pombe *Orb6 [45] and interacts with MOB1 to form an active kinase complex that has a potential role in cytokinesis, but not mitosis [46]. Whether the conserved NDR kinases also interact with MOB1 is not yet known. The remainder of the AGC kinases could not be assigned to a specific family by sequence alone, except for one RSK-like sequence. Phylogenetic inference of the *T. brucei *sequences carried out as detailed in Methods supports these general conclusions and provided no indication of trypanosomatid-specific clusters (data not shown).

#### Over-represented groups: CMGC and STE

The CMGC group and the STE group are relatively well represented within these trypanosomatid genomes as compared to humans. Examples of CMGC kinases include ePKs such as cyclin-dependent kinases (CDKs), MAP kinases (MAPKs), and dual specificity CLK and DYRK kinases. Trypanosomatids have a large number of these kinases (e.g., 45 in *L. major *as compared to 61 in humans). All of the CMGC families identified in humans are also represented in trypanosomatids, as indicated by BLAST analysis and phylogenetic inference. The CDK family is relatively large in trypanosomatids with 11 members in *T. brucei *and *L. major *and 10 in *T. cruzi*. This complexity may reflect the problem of dividing a highly polarized cell with an elaborate cytoskeleton and a single mitochondrion, along with an integral link between cell cycle control and life cycle differentiation. Despite the existence of a large number of CDK family members (named CRK for cdc2-related kinase), only 2 have been shown to be essential for cell cycle progression in trypanosomatids. CRK3 in complex with the CYC6 mitotic cyclin is essential for G2/M phase progression and is the functional homologue of CDK1 [36-38,47]. CRK3 in complex with CYC2 is essential for G1 progression [48,49]. A PHO80-like cyclin and a B-type cyclin control the cell cycle of the procyclic form of *Trypanosoma brucei *[50], while *TbCRK1 *is also an essential gene required for G1 phase progression [38,47,51,52]. However, the roles of CRKs in the cell cycle is complex, with functional differences between bloodstream and procyclic form *T. brucei *as revealed by RNAi knockdown studies [37,38,47,48]. CRK7 has the highest level of sequence identity to CDK7 of mammals. CDK7, in complex with cyclin H and MAT1, is a CDK-activating kinase (CAK) that phosphorylates the T-residue of CDKs (e.g., T160 of human CDK1). No cyclin H or MAT1 orthologues can be identified in trypanosomatids based on sequence, so it remains to be determined if CRK7 is a functional cyclin-dependent kinase or indeed if it has CAK activity. However, many CRKs, including CRK1, 2, 3, 6, 7, 8, 9 and 12, have a conserved T-loop residue, suggesting that the CRKs might be activated *in vivo *by a CAK activity [53].

Interestingly, *T. cruzi *possesses a large number of genes encoding CRK7 isoforms (counted only as one unique gene in our analyses). These are dispersed near the telomeres of many chromosomes, being adjacent to a retrotransposon hotspot protein gene. Of the 27 sequences identified, most contain all of the catalytic residues, although a few are truncated. The biological significance of this gene amplification is not known, however expansion of gene families within subtelomeric regions of trypanosomatid chromosomes is a feature of these genomes in general.

Two families of CMGC kinases phosphorylate serine/arginine rich motifs in serine-arginine rich SR proteins, which function in RNA processing and splicing in many higher organisms: SRPKs [54] and the dual specificity CLKs [55]. Two SRPKs [54] and four or five CLKs are encoded within each trypanosomatid genome. Given the major role of RNA processing and turnover in modulating gene expression in trypanosomatids, these families of CMGC kinases may be of key interest in studying parasite gene regulation. Two GSKs, which are drug targets in diabetes and neurological diseases [56], are also present.

A large number of MAPK-related genes are also present in trypanosomatids, possibly reflecting a role of 3-component MAP kinase cascades in coordinating responses to environmental cues (Table 1). Among these genes are those which are most closely related in sequence to the MAPK family, and those which are most closely related to the CDKL and RCK families. These latter families possess the residues characteristic for the regulation of MAPKs and hence are considered to be part of a MAPK superfamily by some authors [57], even though they are more similar in sequence to CDKs. Among the identified MAPK-like predicted proteins, two sets lack the predicted regulatory motifs (LmjF13.0780 and its *T. cruzi *orthologue; LmjF03.210 and its *T. brucei *and *T. cruzi *orthologues, Table S1) and hence must be regulated in a distinct manner. Thus the total complement of protein kinases likely to be regulated as MAPKs numbers 14 in *T. brucei*, 13 in *T. cruzi*, and 15 in *L. major*.

The parasites clearly find themselves in environments that vary substantially in temperature, pH, nutrients, and stresses during their developmental cycle. An elaborate phosphorylation signaling system to respond to those changes may be a key strategy of this group of organisms. Many MAPKs, and those kinases likely to regulate them (see below), appear to be involved in developmentally regulated processes in trypanosomatids. Nine MAPKs have been cloned and analyzed from *L. mexicana *(LmxMPK1-9) and their mRNAs abundances are developmentally regulated [58,59]. LmxMPK1 is essential for amastigote, but not promastigote proliferation [59], while LmxMPK9 is involved in regulating flagellar length, a stage-regulated function in *Leishmania *[60]. Three MAPKs have been analyzed in *T. brucei*. KFR1, an ERK-like MAPK, has been proposed to be involved in the proliferation of bloodstream form trypanosomes and is the first trypanosomatid ePK reported to be regulated by a specific extracellular molecule, interferon γ [61,62]. TbMAPK2, also ERK-like, is not essential for proliferation of the bloodstream form trypanosome, but is important for successful differentiation [63]. Mutants lacking TbMAPK2 have delayed kinetics of differentiation from the bloodstream form to the procyclic form; the resulting procyclic forms undergo cell cycle arrest. TbECK1, which has characteristics of both MAPKs and CDKs, and was named *T. brucei ***E**RK-like, **C**DK-like protein **k**inase [64], falls into the CDKL family by phylogenetic analysis (this study). This kinase appears to be essential in all life cycle stages analyzed [64]. TbECK1 has an unusual C-terminal extension and overexpression of TbECK1 lacking the C-terminal extension in procyclic trypanosomes leads to a significant reduction in growth, suggesting an important role in cell cycle control. The C-terminal extension appears to act as a *cis*-acting negative regulator of protein kinase. The roles of many trypanosomatid MAPKs remain to be explored.

**Figure 5:**
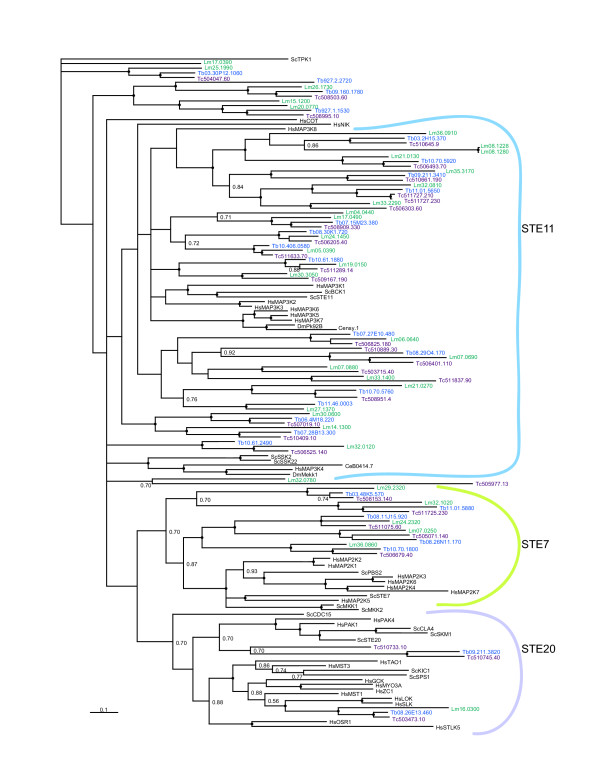
**Phylogram of STE kinases**. Kinase domains from the TriTryp predicted proteins classified as STE by BLAST or STE-like from the *T. brucei *tree in Figure 2 were analyzed using MRBAYES. Also included are STEs from all families present in humans (Hs), plus examples from *S. cerevisiae *(Sc), *C. elegans *(Ce) and *D. melanogaster *(Dm). Nodes with a bootstrap value greater than 0.95 are marked by a dot, while values ranging from 0.7–0.95 are indicated numerically. Similar results were obtained with PAUP*. Gene names are shown as in Figure 4. ScTPK1, an AGC kinase, used as an outgroup.

MAPKs are activated by phosphorylation within the activation loop, typically both on a tyrosine and a threonine. This phosphorylation is mediated by MAP kinase kinases (MAP2Ks), which are members of the STE7 family, one of the three major families of STE group kinases that are generally described as upstream regulators of MAP kinase cascades. Although only two STE7 genes were assigned through BLAST analysis, phylogenetic inference revealed that five sets of orthologues cluster with good confidence into the STE7 family (Figure 5), suggesting that they may function as MAP2Ks in this organism. A previously identified *Leishmania mexicana *MAP2K, LmxPK4, has a potential role in parasite differentiation [65], while another LmxMKK, is involved in the maintenance of flagellar length [66]. STE11 family ePKs often function as MAP3Ks and are especially numerous in the trypanosomatids. Several of the STE11 kinases formed trypanosomatid-specific clusters in our phylogenetic analyses. Another cluster was found to be ubiquitous amongst the trypanosomatid, yeast and human kinomes. LmMRK1 (LmjF32.0120) is an essential STE11 family kinase [67]. In contrast to STE11, the STE20 family kinases, many of which function as MAP4Ks, are relatively rare in trypanosomatids. Another arm of the MAPK activation pathways is mediated by RAF1, a TKL kinase. The TKL group of protein kinases is absent in trypanosomatids. It is clear that sequence data alone cannot accurately predict specific three-component signaling pathways in the trypanosomatids – detailed biochemical analyses will be required. Nonetheless, taken together, these findings provide interesting insight into trypanosomatid-specific aspects of MAP kinase cascades. The STE group of kinases is relatively expanded in trypanosomatids, with 34 members in *L. major*. In contrast, it is either absent or highly abbreviated in the malaria parasite [30,31], once again highlighting the differences amongst protozoan lineages.

#### Other serine/threonine kinases

**Figure 6:**
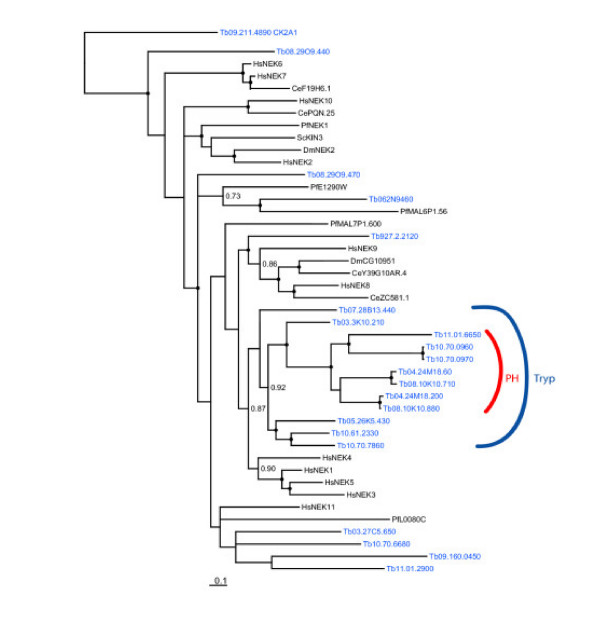
**Phylogram of NEK kinases**. NEK sequences from human (Hs), *S. cerevisiae *(Sc), *C. elegans *(Ce), *D. melanogaster *(Dm), *P. falciparum *(Pf) and *T. brucei *(Tb) were analyzed using MRBAYES. TbCK2A1 was used as an outgroup. Nodes with a bootstrap value greater than 0.95 are marked by a dot, values ranging from 0.7–0.95 are indicated numerically. Similar results were obtained with PAUP*. Two NEK kinases had recognizable PH domains that did not achieve the Pfam HMM search cutoff (Tb04.24M18.60, and Tb08.10K10.710).

The NEK family of ePKs shows a significant expansion within trypanosomatids, having 20–22 members (compared to the 15 representatives in the human genome). The NEK kinases have been relatively little studied in model systems, but several appear to be involved in cell cycle [68] and cytoskeletal functions [69,70]. Some of the NEK kinases appear to function in cascades, with human NEK9 phosphorylating and activating NEK6 and NEK7 [71]. Indeed, all of the *T. brucei *NEK kinases possess the RD motif in subdomain 6, which is an indicator that phosphorylation in the activation loop is likely to be required for maximal activity. As with most of the human NEK kinases, the catalytic domain is situated at or near the N-terminus of the *T. brucei *NEK kinases. Phylogenetic analysis of the 20 *T. brucei *NEK kinases shows that the parasite kinases do not form tight clusters with the NEK kinases represented in the 4-kinome database nor with the NEK kinases of the protozoan *P. falciparum *(Figure 6), although in two of three phylogenetic methods implemented (MRBAYES and PHYML Likelihood), one of the *T. brucei *NEK kinases (Tb06.2N9.460) did cluster with a plasmodial kinase (MAL6P1.56). In contrast, several clusters of NEK kinases across yeast and metazoa were identified: e.g., ScKIN3, DmNEK2 and HsNEK2 form a highly supported clade, as do HsNEK10 and CePQN25. Of particular interest to us was the identification of a trypanosomatid-specific clade containing 12 of the *T. brucei *NEK kinases, which was supported by all of the methodologies. The trypanosomatid NEK kinases have perhaps a modestly higher preponderance of accessory domains compared to other trypanosomatid kinases (see below). For example, several possess a coiled coil region downstream of the catalytic domain (Tb03.27C5.650, Tb05.26K5.430, Tb10.61.2330, and Tb10.70.7860). This feature is also found in human NEK1 and NEK2. Several other trypanosomatid NEK kinases have a C-terminal PH domain, a combination not described in the NEK kinases of other species. These kinases lie within the trypanosomatid-specific clade. The roles of the trypanosomatid NEK kinases have not been studied in any detail, although at least one is known to be developmentally regulated [72] and one has a role in basal body duplication (D. Robinson, personal communication).

Among the families of "Other" protein kinases represented in trypanosomatids, several have been shown to be involved in cell division in various organisms (AUR, Aurora; PLK, polo-like kinases; Wee1) DNA replication/repair (TLK, also ATM/ATR atypical kinases described below), and stress responses (PEK family). Activators of CAMKs (CAMK kinases, CAMKK) are also present, as are multiple CK1 and CK2 isoforms (formerly known as casein kinase I and II) [73,74]. A member of the VPS15 family (involved in **v**acuolar **p**rotein **s**orting), was also identified, although the *Leishmania *orthologue may not be catalytically active. One representative of the ULK family kinases was found in each trypanosomatid. ULK kinases are involved in autophagy in yeast [75] and in pattern formation and development in multicellular organisms [76].

**Figure 7:**
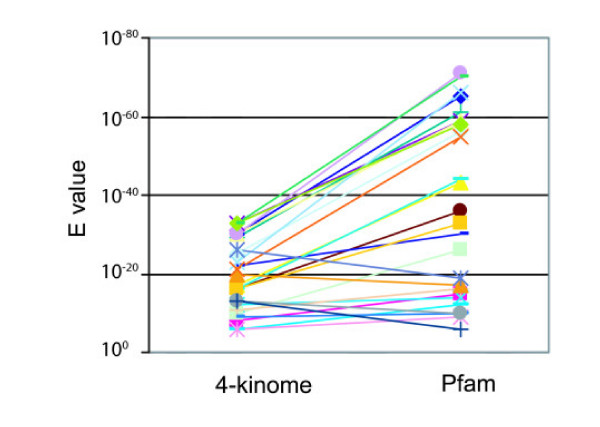
***T. cruzi *unique ePKs: similarity to other ePKs and Pfam kinase domain**. The E-values for the relationship of individual T. cruzi ePKs as compared to the 4-kinome dataset and to the Pfam domain. Many of the T. cruzi ePKs show strong E-values against the Pfam kinase domain, despite their low similarity to the kinases in human, *D. melanogaster *a, *C. elegans*, and *S. cerevisiae*.

A significant number of ePKs were classified as unique, as they showed no clear affinity to any known group or family within the 4-kinome dataset. For example, a number of *T. cruzi *ePKs which had significant matches to the protein kinase Pfam domain signature (Pfam 00069) did not show any distinct similarity to specific kinases in the 4-kinome dataset. Of this group, half had E-values of 10^-35 ^or better against the Pfam domain (Figure 7). On the other hand, approximately one-third of the *T. cruzi *unique kinases showed relatively poor matches against the Pfam domain (E-values ≤ 10^-16^), but nonetheless were observed to possess a complete subdomain structure as well as the required catalytic residues. The ePKs classified as unique were the least conserved among the trypanosomatids, with 63% being absent in at least one of the three species. As such, the unique kinases are likely to represent instances of lineage-specific evolution defined by gene gain and/or loss in these organisms. Such divergent kinases may provide a set of useful protein kinase drug targets, since they have no closely related homologues in the host.

### Membrane kinases interfacing with the environment?

Most mammalian receptor kinases belong to the tyrosine kinase group, a group which is lacking in trypanosomatids. However, in plants, most receptor kinases are serine/threonine kinases. Bearing this in mind, we searched the *T. brucei *genome for genes bearing the protein kinase Pfam domain plus the annotation of a transmembrane domain. Ten candidates fit the criteria (see Additional file 3), these were spread among a variety of ePK groups, with a somewhat higher representation among the STE kinases. At this juncture, there is no evidence that any domain of these molecules is displayed on the parasite surface, where it might respond to host or parasite derived ligands. Alternatively, if surface-localized, the kinase could phosphorylate host or parasite molecules to modify their environment. We note with interest previous reports of an ectokinase with a substrate profile characteristic of CK1 in *Leishmania *[77,78]. Intriguingly, one of the *L. major *CK1 genes identified in this analysis encodes a protein with a predicted signal anchor sequence (LmjF17.1780). Assessing whether any parasite protein kinases interface with the host environment is an important arena for future experimental studies.

### Inactive protein kinases

Approximately 8% of the ePKs of each species are predicted to be catalytically inactive, based on the presence of mutations in essential residues (K in subdomain 2 and D in subdomains 6 and 7). Most of these possess an orthologue in at least one other trypanosomatids. Of the 13 *T. brucei *ePKs predicted to be catalytically inactive, 11 are mutated to a predicted non-catalytic form in each of the three species. Genome-wide, the level of amino acid sequence identity among COG members averages 61 +/- 7% between *T. brucei *and *T. cruzi *[79], with a similar level of identity for a sampling of ePKs (60% +/- 7%). The ePKs predicted to be inactive show a lower level of identity at 44% +/- 8%. Hence, while conserved, these sequences are somewhat more divergent across species.

We also estimated the synonymous (Ks) and nonsynonymous nucleotide (Ka) nucleotide substitution rates in *T. brucei *versus *T. cruzi *genes encoding ePKs predicted to be catalytically active or inactive. The Ka/Ks ratio (sometimes designated as dN/dS) can reflect the selective constraints on a gene. Ka/Ks = 1 is expected for genes evolving neutrally. Ka/Ks < 1 is thought to indicate selection to remove amino acid replacements. In the rare cases where the Ka/Ks > 1, selection for amino acid divergence is usually invoked. For a random subset of 90 active ePKs, Ka = 0.336, Ks = 4.822 and Ka/Ks = 0.077. For the inactive ePKs, these figures were 0.535, 9.639, and 0.110, respectively. These data indicate that in both sets synonymous mutations are highly preferred. Nonetheless, the Ka and Ka/Ks were significantly different in the active versus inactive datasets (p = 0.0003 and p = 0.0045, Mann-Whitney U-test, two tailed). There was no statistically significant difference in the calculated Ks scores between these two datasets (p = 0.3). These findings suggest that the encoded proteins continue to play a significant functional role within the organisms, although the predicted lack of catalytic activity indicates this role is likely to be via a distinct mechanism, such as regulation via protein-protein interaction. Indeed, a recent analysis has shown that inactive protein kinases are not an exception in metazoa and that a few have evolved novel functions, some of which might be involved in processes that enhance the complexity of regulatory phosphorylation networks [80].

### Accessory domains

A characteristic of human ePKs is the presence of accessory domains. Indeed, over 50% of human protein kinases have additional Pfam domains, and more are found when criteria are relaxed [21]. We examined all of the trypanosomatid ePKs for significant matches to additional Pfam domains (Table 2). In the case of *L. major*, only 25 ePKs possessed additional Pfam domains that met the default cutoff. Three additional ePKs, which had orthologues in *T. brucei *or *T. cruzi *that had a significant Pfam domain, were found to possess partial motifs, and others had domains of lower significance. The accessory domains were generally conserved among members of a COG. Notably four of the five most common Pfam domains on human ePKs are absent in the trypanosomatid kinome: Ig, fn3, SH2, and SH3. Ig and fn3 domains are generally extracellular domains that interact with ligands, so their absence may not be surprising given the paucity (or absence) of receptor ePKs in trypanosomatids. SH2 domains interact with phosphotyrosine, and their absence in the trypanosomatid genomes could suggest a co-evolution with dedicated tyrosine kinases. SH3 domains bind to proline rich sequences.

**Table 2:** Additional Pfam domains on trypanosomatid ePKs

**Pfam**	**Tb^a^**	**Tc**	**Lm**	TriTryp^b^	Other kinomes^b^	Comments
Armadillo	0	1	1	ULK	NEK, STE11, ULK	related to HEAT
B-box Zn finger	0	0	1	NEK	nd	Zn binding
C1-like	0	1	1	AGC	AGC	possible diacylglyerol binding
cap-gly	1	1	1	CAMK	VPS15	cytoskeleton associated
cNMP binding	0	1	2	STE	AGC	cyclic nucleotide binding
EF Hand	2	2	2	CAMK	CAMK	Calcium binding
FHA	1	2	4	CAMK, STE	CAMK	phosphopeptide binding
FYVE	1	2	1	AGC	nd	Zn binding
HAMP	0	1	0	STE	nd	in diverse signaling proteins
HEAT	1	1	0	ULK	VPS15	protein interaction
HECT	0	0	1	unique	nd	ubiquitin transferase
Kelch	1	0	0	unique	nd	propeller structure
LRR	1	1	1	CAMKK	TK, TKL, plantrk	protein interaction
MORN	1	1	1	STE	TKL, unique	unknown function
PAS	1	1	1	STE	CAMK, TKL	signal sensor
PH	6	7	6	AGC, NEK	AGC, CAMK, STE20	Phosphatidylinositol binding
PKC-Cterm	0	0	2	AGC	AGC	found on protein kinases
POLO	1	1	1	POLO	POLO	POLO kinase region
PX	1	1	1	AGC	AGC	phosphoinositide binding
RWD	1	0	0	PEK	PEK	unknown function
TPR2	0	2	1	STE, unique	plantrk	protein interaction
zf-C2H2	2	1	1	unique	AUR	zinc binding

^a ^The number of genes bearing the indicated Pfam domain.

^b ^Classification of ePKs bearing the designated domain in the TriTryps or other kinomes (as detected in *S. cerevisiae*, *Schizosaccharomyces pombe*, *P. falciparum*, *C. elegans*, *D. melanogaster*, *A. thaliana*, and *Oryza sativa*) using the web tools at the Kinases in Genomes (KING) website [81]. The plantrk group is comprised of serine/threonine receptor kinases present in plant; nd, none detected.

**Figure 8:**
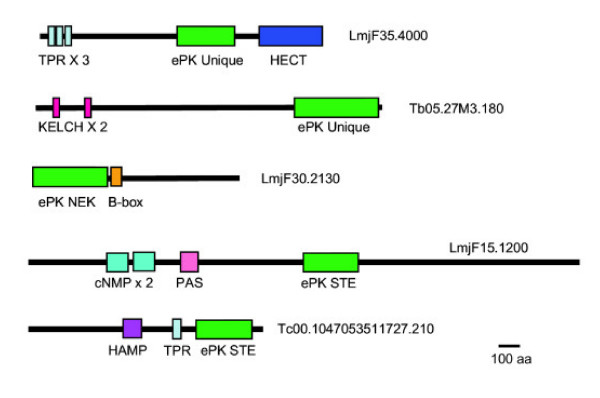
**Domain structure of Tritryp ePKs with unusual additional domains**. Examples from Table 2 are shown. ePK domains are predicted to be active.

Several unusual domain combinations are found in trypanosomatid protein kinases (Table 2, examples shown in Figure 8). A search of eight eukaryotic kinomes using the KinG Kinases in Genomes Resource [81] revealed that some accessory domains found in trypanosomatids that were not associated with ePK catalytic domains in other species. For example, LmjF35.4000, which is a *Leishmania*-specific gene, contains a unqiue ePK catalytic domain, along with three TPR motifs (which are present on some plant receptor kinases) along with a domain associated with ubiquitin transferase (HECT), a domain not seen in the sampled genomes. A *T. cruzi *ePK, Tc00.1047053511727.210, has a TPR motif and HAMP domain (found on various signaling proteins including histidine kinases), both of which are recognizable on the *T. brucei *orthologue, but not on the *L. major *orthologue. Others domains are associated with ePKs of different classification. For example, the very large STE kinase LmjF15.1200, has an unusual juxtaposition of two domains related to cyclic nucleotide binding and a PAS domain, associated with signal sensing. In other species, the PAS domain is found on CAMK and TKL kinases, while the cNMP domain is restricted to AGC kinases. The *L. major *domain structure is likely conserved in *T. cruzi*, although the coding region is interrupted by a contig break. No orthologue is present in *T. brucei*. Some other accessory domains are found on similar groups of kinases (RWD, PX). Despite the paucity of identified domains, the trypanosomatid ePKs are generally considerably larger than the 250 aa kinase domain. For example, half of *T. cruzi *ePKs are larger than 64 kDa, and 38 are larger than 100 kDa.

### Atypical protein kinases

**Figure 9:**
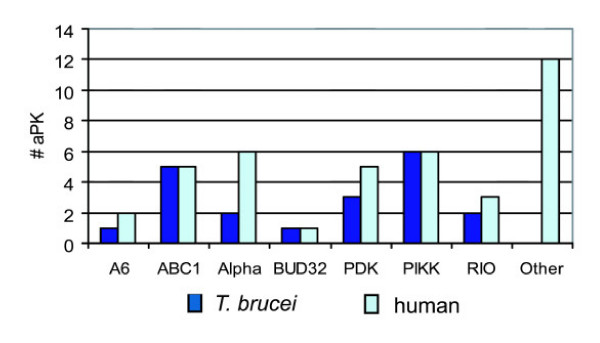
**Comparison of *T. brucei *and human atypical protein kinase classification**. See Additional file 4 for systematic gene names.

The parasites possess a complement of atypical protein kinases, including representatives of all of the more well-characterized families: RIO, alpha, PIKK and PDK (Figure 9 and Additional file 4), although no functional analyses have been carried out to date on any representative aPK from trypanosomatids. The RIO family of atypical kinases is related to ePKs, but RIO proteins lack the sequences known to be involved in peptide binding in ePKs [82]. Nonetheless, the catalytic residues are present. Trypanosomatids possess two RIO proteins, which are clearly assigned to the RIO1 and RIO2 subfamilies. In other organisms both RIO1 and RIO2 are required for ribosomal biogenesis, and RIO is involved in cell cycle progression [26]. Interestingly, the similarity between human and trypanosomatid RIO2 extends into the N-terminus, where the structure of the human enzyme shows a winged helix-turn-helix motif [82]. Such helix-turn-helix motifs are often found on DNA binding proteins such as transcription factors, a class of proteins which are rare in trypanosomatids.

The alpha kinases, so named because they phosphorylate their substrates within alpha helices, show a small amount of sequence similarity to ePKs, with conservation of the catalytic residues in subdomains 2, 6, and 7 [27]. One set of the alpha kinases in trypanosomatids is comprised of small molecules, being little more than the 241 aa alpha domain. This type of alpha kinase is present in all three species. However, *L. major *possesses two additional alpha kinase genes, which are very large (>1000 amino acids). Interestingly, three of the four *L. major *alpha kinases are found on a 12 kb segment on chromosome 36. None of the alpha kinases appear to be fused to an ion channel, as is the case for certain vertebrate alpha kinases [27].

The PIKK kinases represent a particularly interesting family in which the protein kinase domain structurally resembles that of phosphatidylinositol 3-kinases [25]. In addition to the kinase domain, these proteins also have FAT and FATC motifs which are not found in the lipid kinases. The similarity between the trypanosomatid PIKK kinases and those in the 4-kinome dataset is highly significant, with E-values of 10^-90 ^or better. PIKK kinases are quite large in general and those in *T. brucei *are no exception, ranging in predicted size from 271 to 468 kDa. The parasites possess clear homologues to the specific PIKK kinases involved in genome surveillance: ATM and ATR [83]. They also have four kinases that belong to the FRAP family (this family includes FRAP and mTOR). TOR (target of the immunosuppressive agent rapamycin) modulates translation and cell cycle in response to nutrient and growth signals [84]. Multiple drugs targeting mTOR are in trials for the treatment of various cancers [84].

The trypanosomatids contain 3 genes encoding putative pyruvate dehydrogenase kinases (PDK). In mammals, the activity of mitochondrial pyruvate dehydrogenase is tightly regulated by multi-site serine phosphorylation of the E1α subunit [85]. Despite their exclusive phosphorylation of serine residues, the PDKs lack the domains characteristic of ePKs. Rather, these kinases have two distinct domains. A C-terminal domain that shares structural conservation with the GHKL ATPase/kinase superfamily (including members of the histidine kinase family) and an N-terminal domain that resembles a histidine phosphoryl transfer domain of bacterial two component systems [86]. The presence of an active pyruvate dehydrogenase in trypanosomatids with an E1α subunit suggests that regulation of activity by phosphorylation is likely to be conserved in these species.

## Conclusion

The analysis presented here shows that trypanosomatids possess a large complement of protein kinases, indicating that protein phosphorylation is a key mechanism for regulation of parasite processes. In metazoa and yeast, the ultimate targets of many signaling cascades are transcription factors, which then trigger the expression of new sets of genes. In contrast, since trypanosomatids indiscriminately transcribe most genes in large polycistronic units, signaling cascades in these organisms must function in post-transcriptional regulation. Key regulators of specific mRNA turnover are still being sought, and we propose that protein kinases are major players in these processes. We also propose that trypanosomatids, more than many other organisms, rely on the phosphorylation of the downstream molecules that perform stage-specific and cell-cycle specific functions. Phosphorylation has been shown to modulate protein turnover, localization, interaction and activity for various molecules in eukaryotes. Both ePKs and aPKs are the targets of major drug discovery efforts in chronic human diseases [56,84,87,88]. Exploiting the knowledge and resources generated in those efforts could provide new answers in the search for new drugs to combat trypanosomatid diseases. A major effort to understand the functions of individual protein kinases will allow increased focus on key molecules. We suggest that PKs closely related to human drug targets would be a useful first set to be explored. However, perhaps just as useful could be the group of unique kinases, which show little resemblance to human PKs.

## Methods

### Classification

All ePKs were retrieved from GeneDB [29] through a combination of searches with the protein kinase Pfam domain, BLAST analysis using diverse ePKs, and examination of COGS. *L. major *(version 5.0, Feb 2005); *T. brucei *(version 4.0, Feb 2005) and *T. cruzi *(version 3.0, July 2004) were the final datasets used in this study. In the case of *T. cruzi*, in which the genome strain is a hybrid, the two presumed alleles were identified through analysis of COGs [79], and counted as one gene, even though up to 7% sequence allelic sequence divergence occurs in this strain [5]. All ePKs were examined for the presence of the 11-subdomain structure, and the presence of lysine in subdomain 2 and aspartic acid in subdomains 6 and 7, which are required for catalysis [24,89]. Those lacking these residues were categorized as catalytically inactive. Similarly, a few ePKs lacked any sequence resembling subdomain 1, which functions in ATP binding, and were also categorized as catalytically inactive. Atypical PKs were identified by BLAST analysis using representatives of each group of aPKs from other species as queries.

Each ePK was analyzed for the presence of additional domains by hidden Markov model analysis of the Pfam database [90]. The default cutoffs were used.

All predicted ePKs from each species were analyzed by BLAST analysis against the 4-kinome dataset comprised of all human, *Saccharomyces cerevisiae*, *Drosophila melanogaster*, and *Caenorhabditis elegans *protein kinase domains [34]. Since phylogenetic inference indicated that members of trypanosomatid COGs were more closely related to each other than to ePKs of the 4-kinome dataset, all COG members were classified congruently (the sole exception we found was two kinases that shared extensive homology outside of the kinase domain, LmjF15.1200 and Tc00.1047053505977.13). Assignments required an E-value difference of 5 logs or more between groups or families of ePKs for one of the trypanosomatid orthologues. When all trypanosomatid orthologues had E-values poorer that 10^-16^, or had similar E-values for different groups of ePKs, those ePKs were designated "unique".

### Phylogenetic inference

Due to the relatively low degree of sequence conservation at the nucleotide level within some of these families, phylogenetic inference was carried out on amino acid alignment, rather than attempting alignment at the nucleotide level. Kinase domains were identified by analysis of alignments with the Pfam protein kinase domain, and extended manually as needed. Insertions larger than 25 amino acids were identified and removed prior to subsequent analyses. The SAM (Sequence Alignment and Modeling System using Hidden Markov Model (HMM) software was used to build HMMs representing the kinase domains of the gene families discussed in this paper [91]. These trained models were then used to identify residues capable of discriminating between the various domain families. In addition, by aligning the sequences of the kinase domains to these models, we created multiple sequence alignments of these gene families. These alignments were then visually inspected to verify that all subdomains were appropriately aligned, as well as to allow removal of both gene-specific insertions (in addition to those previously removed) and deletions and phylogenetically uninformative residues. These edited HMM-generated alignments were used as the starting point for phylogenetic reconstruction of these domain families.

Phylogenetic analysis of the kinase domains of these proteins was carried out using a variety of techniques. As a preliminary step in the phylogenetic investigation of our dataset, we used the Neighbour-joining approach as defined by Saitou and Nei as implemented in ClustalX [92]. Due to the relatively high degree of divergence that might be expected within our dataset we used the correct for multiple substitutions option in our analysis. Bootstrapping was carried out on the dataset with 1,000 replicates.

These aligned amino acid sequences were also subjected to parsimony analysis using PAUP*, version 4.0b8 [93,94]. Given the relatively large number of taxa in this dataset, the use of an exhaustive search was not possible. In its place, an heuristic search strategy was employed to attempt to find the best tree by reducing the set of trees examined and just calculating the score for likely trees. It should be noted that this method is not guaranteed to identify the most parsimonious trees from the sequences. We carried out 100 random stepwise addition sequences of taxa, each with TBR swapping and MAXTREES set to 10,000. Parsimony bootstrapping on the dataset was performed with 1,000 replicates and the same settings, except that only 10 random stepwise addition sequences were used per bootstrap replicate.

The same datasets were analysed using a Maximum Likelihood approach, as implemented in the web available PHYML application [95,96]. Analysis using the WAG amino acid substitution model inferred the starting tree. The proportion of invariable sites and the gamma distribution parameter were estimated by maximizing the likelihood of the phylogeny. The number of substitution rate categories was set at four for these analyses. Non-parametric bootstrap analysis was then carried out on the original data set with 500 replicates (the upper limit available for this web service). Majority-rule consensus trees were created for each of the three methodologies outlined above.

Finally, MRBAYES was used to carry out a Bayesian analysis of our data [97]. We used the WAG amino acid substitution model (based on nuclear genes and globular protein sequences, respectively), with a gamma rate distribution estimated from the data set to infer the phylogeny of our dataset. Starting from random trees, four parallel Markov chains were run to sample trees using the Markov Chain Monte Carlo (MCMC) principle. In general, 1,000,000 generations were run; after the burn-in phase, every 100^th ^tree was saved.

### Estimation of Ka/Ks

Analysis of the synonymous/non synonymous substitution rates required construction of pairwise, codon aligned, sequence alignments. These were generated using the PAL2NAL web server [98], which converted full-length amino acid alignments and the corresponding DNA sequences into a codon-based DNA alignment. The amino acid sequence alignments were obtained using the Water programme from the EMBOSS package using default parameters [99]. Estimation of Ka/Ks (dN/dS) ratios was then carried out by maximum likelihood using the pairwise codon-based substitution model in Codeml, which is part of the Phylogenetic Analysis by Maximum Likelihood (PAML) suite of programs [100].

## List of abbreviations

aPK, atypical protein kinase; COG, clustered orthologous groups; ePK, eukaryotic protein kinase, PK, protein kinase, TriTryp, the trypanosomatids *Leishmania major*, *Trypanosoma brucei*, and *Trypanosoma cruzi*.

## Authors' contributions

MP carried out the analysis of *T. brucei *and *T. cruzi *protein kinases and drafted the manuscript. EAW performed the phylogenetic inference analyses. PNW carried out the analysis of *L. major *protein kinases. JCM contributed to interpretation of the data and the writing of the manuscript. MP and JCM conceived and coordinated the study. All authors read and approved the final manuscript.

## Supplementary Material

Additional file 1 Table S1, Classification, orthology and systematic IDs of trypanosomatid ePKs Click here for file

Additional file 2 Table S2, *T. brucei *protein kinases with predicted tyrosine in subdomain I Click here for file

Additional file 3 Table S3, *T. brucei *protein kinases with predicted transmembrane domains Click here for file

Additional file 4 Table S4, Classification, orthology and systematic IDs of trypanosomatid atypical PKs Click here for file
